# On the fragility of neural architecture search: the role of overfitting and task complexity in medical image analysis

**DOI:** 10.3389/frai.2026.1869820

**Published:** 2026-07-22

**Authors:** A. Gómez, M. Desco, M. Abella

**Affiliations:** 1 Department of Bioengineering, Universidad Carlos III de Madrid, Madrid, Spain; 2 Unidad de Medicina y Cirugía Experimental, Instituto de Investigación Sanitaria Gregorio Marañón, Hospital General Universitario Gregorio Marañón, Madrid, Spain; 3 Centro de Investigación Biomédica en Red de Salud Mental (CIBERSAM), Instituto de Salud Carlos III, Madrid, Spain

**Keywords:** biomedical image processing, data augmentation, deep learning, ensemble learning, neural architecture search

## Abstract

**Introduction:**

Neural Architecture Search (NAS) effectively automates Deep Learning pipeline design but is prone to validation overfitting when applied to complex tasks, such as medical image analysis. To mitigate this and enhance generalization, researchers frequently integrate Deep Ensemble Learning (DEL) and data augmentation into the NAS workflow. However, the assumption that these methodologies do not negatively interfere in high-overfitting scenarios remains unproven.

**Methods:**

We evaluated NAS, combined with DEL and data augmentation pipelines, across both CIFAR-10 and a biomedical CT dataset, assessing the influence of task complexity and data scarcity in their interaction. Using an ablative experimental design, we isolated the contributions of DEL and NAS and mapped data augmentation sensitivity landscapes at both global and local scales.

**Results:**

While synergistic on large, well defined datasets, statistical analysis revealed that DEL failed to significantly enhance the generalization capabilities of NAS-generated populations in data-constrained regimes. Moreover, we identified local roughness within the data augmentation sensitivity landscapes.

**Discussion:**

Our findings challenge the prevailing assumption of unconditional methodological synergy that guides joint architecture exploration, ensemble pruning and data augmentation optimization.

## 1 Introduction

The vast repertoire of high-performing Deep Learning (DL) architectures has established transfer learning as the standard approach across most research fields. For most computer vision problems, the advantages of bypassing architectural design to focus on data preprocessing and model retraining far outweigh the advantages of creating custom models. However, despite its widespread popularity, transfer learning often fails to yield statistically significant improvements over training from scratch on medical images (Alzubaidi et al., 2021; Guerra-Librero et al., 2023; Luu et al., 2024). The efficacy of knowledge transfer to domains that differ substantially from natural images has long been questioned, with some authors suggesting that observed performance gains stem solely from the over-parameterization of pretrained architectures rather than meaningful feature transfer (Raghu et al., 2019; Neyshabur et al., 2021).

To compensate for suboptimal architectural design, data augmentation is widely employed to strike a balance between generating the highest diversity possible and maintaining sufficient data quality (Mumuni and Mumuni, 2022). However, expanding the data distribution typically necessitates lengthier training, as models must adapt to more complex tasks rather than relying on memorization. More importantly, inadequate transformations can introduce excessive noise, widening the gap between training samples and the real-world distribution (He et al., 2019; Gong et al., 2020). Ultimately, the efficacy of a certain augmentation scheme depends on both the nature of the data and the model’s capacity to capture its nuances. Thus, data augmentation cannot replace appropriate model selection.

Alternatively, Deep Ensemble Learning (DEL) is employed to mitigate suboptimal architectural design by significantly reducing both bias and variance in the final predictions (Ganaie et al., 2022). Similar to traditional ensemble methods (Breiman, 2000), multiple models, referred to as learners, are trained on data subsets to leverage the unique features learned by each one of them. This modularity allows for the use of shallower architectures focused on specific sub-tasks rather than a single, deeper model which, while powerful, is more prone to overfitting. Nevertheless, training multiple models is more computationally expensive than fine-tuning a single architecture. Additionally, achieving learner diversity is difficult when identical architectures share the same inductive biases. Even if the ensembles are composed of diverse architectures, some models may not contribute value or may even degrade overall predictions. Ensemble pruning, also known as selective ensemble methods, is then required to mitigate these negative effects. These methods are based on metrics such as validation error, kappa measure, or diversity (Margineantu and Dietterich, 1997; Martínez-Muñoz and Suárez, 2004; Guo et al., 2017).

Rather than working around suboptimal architecture design, Neural Architecture Search (NAS) is increasingly employed to automate the creation of DL models. By deploying various optimization algorithms across the hyperspace of feasible architectures, NAS has achieved state-of-the-art performance on many computer vision benchmarks (White et al., 2023). However, NAS often requires high computing and time budgets, and the resulting models are prone to overfitting on the dataset partition used for architecture selection. This is inherent to most NAS methods, as it mainly depends on the data distribution and the model selection criteria (Cawley and Talbot, 2010), becoming nearly unavoidable when a vast number of architectures are evaluated. This problem is particularly relevant for complex datasets with limited samples, where constructing representative training and validation partitions is challenging. Unfortunately, using rigorous model selection criteria within NAS pipelines is often infeasible due to compounding computational costs. Furthermore, balancing the exploration-exploitation trade-off is not trivial, especially for very deep networks: additional layers exponentially increase hyperparameter combinations, creating massive search spaces. While the roughness of a model’s weight loss landscape is known to depend on task complexity (Li et al., 2018), recent works demonstrate that the properties of the architectural loss landscape also depend heavily on the nature of the data, both for shallow model clusters (Qiu and Miikkulainen, 2023) and unconstrained NAS runs (Martín et al., 2025). Yet, existing literature (Hernández-García and König, 2018; Balestriero et al., 2022) does not assess how data augmentation perturbs distinct architectural landscapes, but rather reports global observations derived from scarce model sampling on high-n scenarios. Current approaches that optimize augmentation schemes, either standalone (Kang et al., 2025) or alongside NAS (Kashima et al., 2020; He and Chu, 2023), rely on the intuitive premise that similar models share similar inductive biases and benefit similarly from specific augmentations. However, this premise has not been empirically validated. Properly characterizing these shifts would enable more tailored data augmentations and lead to faster, more robust NAS convergence.

Some of the challenges inherent to NAS can be mitigated through DEL, and vice versa. As previously stated, ensemble methods could allow researchers to restrict the search to shallower models, considerably reducing both search space complexity and individual training times without sacrificing predictive power. Deeper models are significantly more expensive to find, not only due to higher training times but because hyperparameter combinations grow exponentially with the number of layers. Furthermore, the characteristic effects of overfitting on model selection partitions may be mitigated by the feature sharing and weighting characterizing DEL, which enhances the generalization capabilities of the combined learners. Conversely, NAS methods inherently maintain populations of solutions with distinct hyperparameters, providing the high diversity that traditional DEL often lacks. Combining NAS-generated models into an ensemble has been shown to surpass the performance of individual learners while being significantly less computationally expensive than searching for a single high-performing architecture. However, the limited literature exploring this approach either focuses exclusively on large benchmarks such as CIFAR-10, ImageNet or COCO (Herron et al., 2020; Chen et al., 2021; Zaidi et al., 2021; Song et al., 2024) or considers only very shallow networks to bypass computational limitations on massive, non-standard datasets (Abulawi et al., 2024). The behavior of NAS-derived ensembles on small, complex datasets, where overfitting is a prevalent issue, remains unexamined. The same is true for the integration of NAS and data augmentation: while a robust data augmentation scheme is expected to enhance NAS performance at the cost of longer convergence times, the precise nature of their interaction has yet to be empirically analyzed.

This work addresses these limitations by carrying out an analysis of the performance of NAS, DEL and data augmentation, both in isolation and in combination with one another, through extensive ablation studies. Our contributions are the following:

- We study the influence of model and data weighting strategies in DEL.
- We introduce the Chimera Algorithm, a novel NAS optimizer focused on maintaining population diversity, and the Gaggle Algorithm, which combines NAS-generated populations into pruned ensembles.
- We provide an initial empirical assessment of how data augmentation influences the architectural loss landscape.
- We assess the synergies and antagonisms between NAS and DEL across both large-scale and low-sample scenarios, showing a real-world use case where the integration of NAS and DEL does not produce significant improvements upon standalone NAS.

Our methodology is first validated on the CIFAR-10 benchmark, comparing various data and model weighting strategies within the DEL framework. We then evaluate these approaches on a biomedical task: predicting detector misalignment in Computed Tomography (CT) systems based on reconstruction artifacts. This allows us to test the performance of our proposed integration of NAS and ensemble learning in a domain where transfer learning is typically the standard approach.

## 2 Materials and methods

In this section, we first introduce the algorithms developed to test our three hypotheses: (1) that the integration of DEL and NAS can mitigate overfitting in small-sample regimes; (2) that architectural loss landscapes are significantly modulated by data augmentation; (3) and that the assumed synergy between these techniques is highly sensitive to the baseline model’s complexity. We then describe the low- and high-n datasets used throughout this work. Finally, we detail the experiments designed to characterize the behavior of DEL and the effects of data augmentation within the NAS framework.

### 2.1 Algorithms

To evaluate the proposed integration of architectural search and ensemble methods, we developed three primary frameworks: a modular Boosting Deep Ensemble (BDE) generator, the Chimera Algorithm (a standalone NAS optimizer), and the Gaggle Algorithm, which combines both to enhance model generalization.

#### 2.1.1 Boosting deep ensemble (BDE) generator

The BDE generator sequentially trains copies of a given base model, weights their contributions to the growing ensemble, and modifies the training dataset according to user-specified strategies. For each new learner, the dataset is either weighted or resampled to prioritize misclassified data. Each learner is also weighted based on its standalone performance, ensuring that the most accurate models exert the greatest influence on the final prediction.

We explored four data-weighting strategies to prioritize samples the ensemble fails to properly characterize: (1) no weighting, (2) resampling the dataset to contain only samples misclassified by the current ensemble, (3) stochastic resampling based on sample weights, and (4) modifying the loss function to incorporate sample weights.

Additionally, we evaluated three model-weighting strategies: (1) uniform averaging of outputs, (2) weighting based on their individual validation performance, and (3) class-specific weighting based on the validation confusion matrix. The algorithm’s pseudocode is shown in Algorithm 1.

ALGORITHM 1 Ensemble generator.
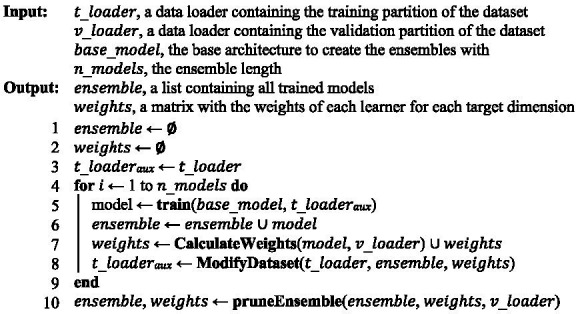

The functions CalculateWeights and ModifyDataset implement the strategies described above. The pruneEnsemble function ranks models by validation accuracy and discards lowest-performing learners (and their corresponding weights) until the ensemble’s overall performance begins to decrease. Sample weights are derived from the AdaBoost framework (Breiman, 2000) as defined in Equation 1:


$$\text{sample}\_\text{weights}=\{\frac{n\cdot w_{i}}{\sum_{j=1}^{n}(w_{j})}|i\in \{1,,\ldots ,,n\}\}$$ (1)

Where n is the number of training samples and $w_{i}$ is the weight for each individual data point. The value for each $w_{i}$ in classification problems is defined in Equation 2:


$$w_{i}=e^{\alpha \cdot \|(y_{i}-p_{i})\|/c}$$ (2)

Where c is the number of classes in the problem, $y_{i}$ is the vectorized binary labels for data point i , $p_{i}$ is the vectorized ensemble’s predicted probability for it to be a positive case for each class, and 𝛼 is a hyperparameter that controls the penalty for misclassification. For regression problems, $w_{i}$ is defined using Equation 3:


$$w_{i}=e^{\alpha \cdot \mathrm{los}s_{i}}$$ (3)

Where 𝑙𝑜𝑠𝑠_i_ is the loss function value for data point i . By default, 𝛼 is set to 1.

#### 2.1.2 Chimera Algorithm (NAS)

The NAS method employed in this work is the Chimera Algorithm, an in-house developed metaheuristic based on the Artificial Bee Colony Algorithm (Karaboga and Basturk, 2007). Unlike genetic or evolutionary approaches, lower-performing models are not discarded at each iteration; instead, they are simply assigned less attention, which has been proven to maintain significantly higher population diversity throughout the search (Kishor and Singh, 2015; Hussain et al., 2020). The novelty of the Chimera Algorithm resides in combining the Artificial Bee Colony search dynamics with the mutation operators from traditional evolutionary NAS, producing diverse architectures that are instrumental in generating high-performing ensembles (Kuncheva and Whitaker, 2003; Bian and Wang, 2007). Its pseudocode is provided in Algorithm 2.

ALGORITHM 2 Chimera Algorithm.
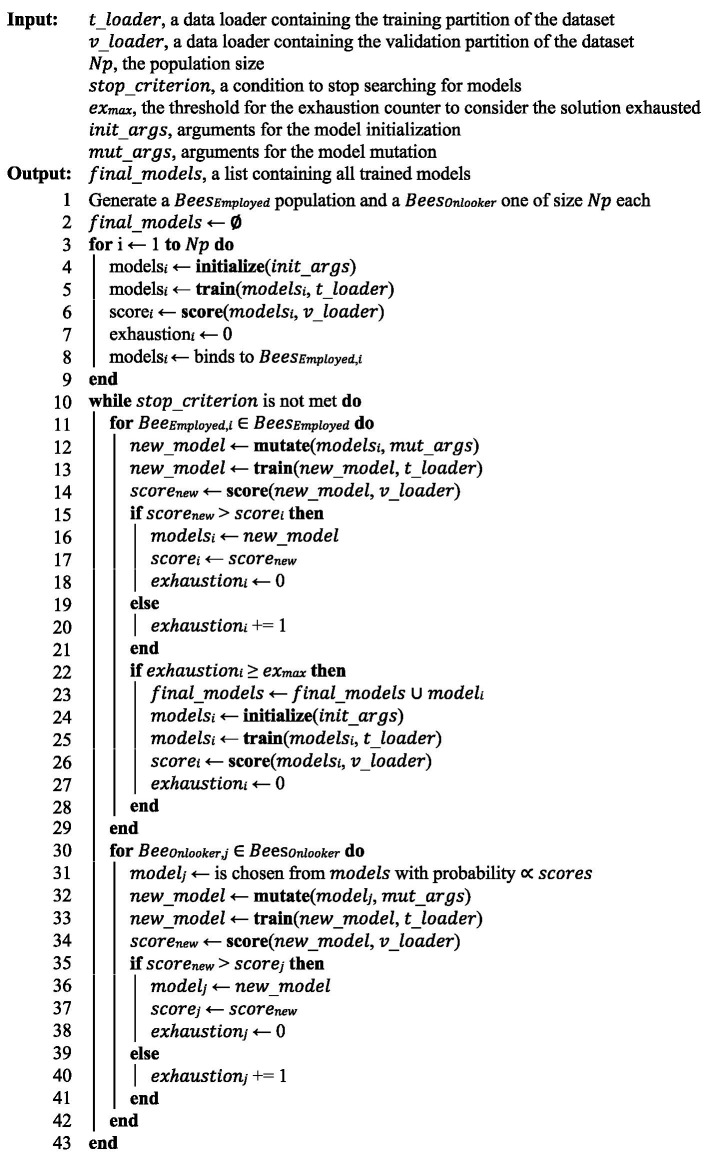

The initial population of architectures is initialized by either copying a provided base model or generating architectures with a random number of layers, depending on 𝑖𝑛𝑖𝑡_𝑎𝑟𝑔𝑠. Once initialized, two types of optimizer agents, 𝐵𝑒𝑒𝑠_𝐸𝑚𝑝𝑙𝑜𝑦𝑒𝑑_ and 𝐵𝑒𝑒𝑠_𝑂𝑛𝑙𝑜𝑜𝑘𝑒𝑟_, explore the hyperparameter space until 𝑠𝑡𝑜𝑝_𝑐𝑟𝑖𝑡𝑒𝑟𝑖𝑜𝑛 is met. 𝐵𝑒𝑒𝑠_𝐸𝑚𝑝𝑙𝑜𝑦𝑒𝑑_ are assigned to specific architectures in a one-to-one mapping (line 8), while 𝐵𝑒𝑒𝑠_𝑂𝑛𝑙𝑜𝑜𝑘𝑒𝑟_ randomly select architectures every iteration with a probability proportional to their validation performance (line 31). Because several 𝐵𝑒𝑒𝑠_𝑂𝑛𝑙𝑜𝑜𝑘𝑒𝑟_ may scrutinize a single promising architecture while others are bypassed, the exploration-exploitation tradeoff naturally favors the most performant regions of the hyperspace.

Both agent types explore around their corresponding architecture by duplicating it and applying mutation operations. An 𝑒𝑥ℎ𝑎𝑢𝑠𝑡𝑖𝑜𝑛 counter tracks each solution, incrementing when a mutation fails to improve the original architecture, and resetting to zero upon success. If a counter exceeds the threshold 𝑒𝑥_𝑚𝑎𝑥_, the architecture is saved as a potential global minimum, and the associated 𝐵𝑒𝑒_𝐸𝑚𝑝𝑙𝑜𝑦𝑒𝑑_ reassigns itself to a newly initialized one (lines 22–28 in Algorithm 2).

Models are trained until convergence to ensure valid performance comparisons. Each model is assigned a fitness score based on the Artificial Bee Colony fitness value, as given by Equation 4:


$$\text{scor}e_{\mathrm{new}}={\left(1+\mathrm{los}s_{v}\right)}^{-1}$$ (4)

Where 𝑙𝑜𝑠𝑠_𝑣_ is the validation loss. This score determines whether to discard mutated models and guides 𝐵𝑒𝑒𝑠_𝑂𝑛𝑙𝑜𝑜𝑘𝑒𝑟_ architecture selection.

The hyperparameters of the Chimera Algorithm are: the search stopping condition 𝑠𝑡𝑜𝑝_𝑐𝑟𝑖𝑡𝑒𝑟𝑖𝑜𝑛 (either a maximum number of iterations or a threshold for the objective loss function), population size 𝑁𝑝, model exhaustion limit 𝑒𝑥_𝑚𝑎𝑥_, initialization arguments 𝑖𝑛𝑖𝑡_𝑎𝑟𝑔𝑠, and mutation arguments 𝑚𝑢𝑡_𝑎𝑟𝑔𝑠. The number of mutations per attempt *n_mut_* is defined in Equation 5:


$$n_{\mathrm{mut}}=\mathrm{int}.(|N(1,\sqrt[{3}]{1+\text{exhaustio}n_{i}})|)$$ (5)

Where $\text{exhaustio}n_{i}$ is the parent model’s current exhaustion value. This ensures fine-grained local exploitation, reserving larger structural jumps for solutions nearing exhaustion. The cube-root transformation considerably limits the magnitude of the structural jumps, which lowers the probability of exploring outside the nearly exhausted solution’s architectural cluster even for high exhaustion limits. By default, architectural mutations (adding, deleting, or modifying layers) each have a 30% probability, with a 10% probability reserved for weight reinitialization without structural changes. To maintain a balanced architecture, the probability of a mutation resulting in a convolutional layer *p_c_* is determined by the current number of convolutional and pooling layers in the model, as defined in Equation 6:


$$p_{c}=\frac{5n_{p}}{\left(5n_{p}+n_{c}\right)}$$ (6)

Where 𝑛_𝑝_ and 𝑛_𝑐_ are current counts of pooling and convolutional layers, respectively. This ensures a ratio of approximately five convolutional layers per pooling layer.

Convolutional and pooling kernel sizes are drawn from a uniform distribution from 1 to 7, and the model length is only limited by the available GPU memory. Layer additions maintain the kernel sizes of surrounding ones. Layer mutations vary the number of output channels from half to twice the original number and modify the next convolutional layer accordingly. Layer deletions modify the input size of the next one.

Stopping criteria and search bounds are the hyperparameters that most significantly affect the quality of the models produced. No default values are provided as they are task-dependent. The other parameters simply define the starting point, preferred direction and average search speed throughout the hyperspace. For instance, (1) keeping a higher probability of removing layers rather than adding them yields a search focused on decreasing model complexity, or (2) using a small population size with a high exhaustion limit favors a thorough exploitation of a few regions rather than widespread exploration. Model exhaustion is deactivated by default. If a base architecture is not provided, the initial population is sampled by randomly mutating a single-convolution model 15 times, producing shallow architectures.

#### 2.1.3 The Gaggle Algorithm

The Gaggle Algorithm integrates NAS into the BDE generator by calling the Chimera Algorithm to generate batches of optimized, diverse models. After every Chimera call, each model in the generated population is assigned a weight and added to the growing ensemble. The ensemble is then pruned, and the dataset is modified according to the pruned ensemble’s performance before subsequent Chimera calls. Its pseudocode is shown in Algorithm 3, with the CalculateWeights, ModifyDataset and pruneEnsemble functions operating as described for the BDE generator (Algorithm 1).

ALGORITHM 3 Gaggle Algorithm.
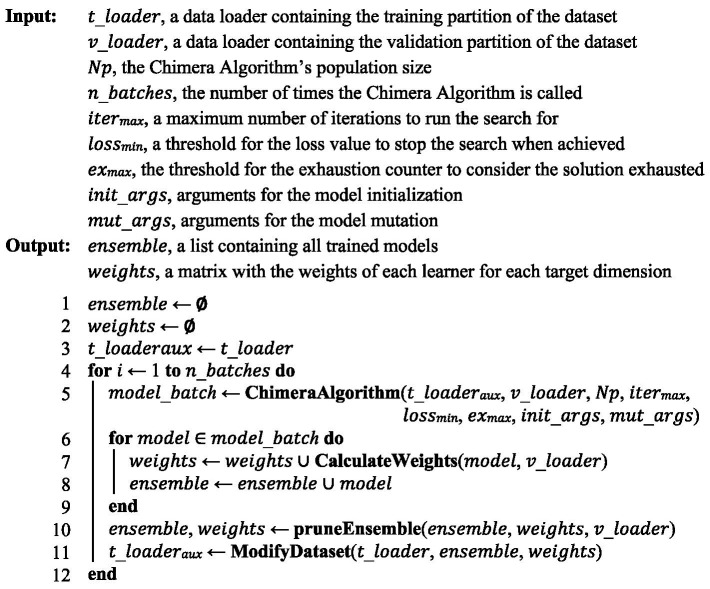

#### 2.1.4 Data augmentation

Finally, we apply standard PyTorch data augmentations to investigate their influence on the architectural loss landscape. The specific set of transformations employed for each task is described in detail in Section 2.3.

### 2.2 Datasets

Two distinct scenarios were considered to investigate varying tendencies toward overfitting during the search and training phases.

#### 2.2.1 Natural image classification (high-n)

In the first scenario, we evaluated our methods using the CIFAR-10 dataset (Krizhevsky, 2009). This dataset consists of 60,000 32 × 32 color natural images across 10 classes. A subset of 50,000 images was partitioned into training (90%, 45,000 images) and validation (10%, 5,000 images) subsets using stratified random sampling with different seeds to generate distinct validation partitions and reduce partition bias. The remaining 10,000 images were reserved as a test split, which was only employed to assess generalization *a posteriori.* Although CIFAR-10 is a standard benchmark for validating deep learning methods, its relatively low complexity and large sample size distinguishes it from most specialized real-world applications.

#### 2.2.2 Computed tomography misalignment (low-n)

**Figure 1:**
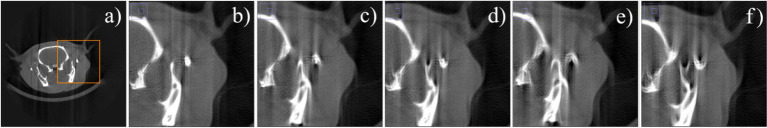
Axial view of a rodent head CT study reconstructed with an angular span of 180° and with no geometric misalignments **(a)**. Detail of the square region reconstructed with simulated horizontal detector misalignments of 0 mm **(b)**, +0.5 mm **(c)**, −0.5 mm **(d)**, +1 mm **(e)** and −1 mm **(f)**.

In the second scenario, we evaluated the methods on a medical imaging regression problem: estimating horizontal detector misalignment in CT based on visual artifacts in the reconstructed 3D volumes. When image reconstruction uses a partial angular span of 180 degrees, these artifacts manifest as arcs in the axial slices (Figure 1). The arc thickness corresponds to misalignment severity, while its orientation indicates the misalignment direction. In practice, these misalignments are typically corrected using periodic scans of a physical calibration phantom. Automatically characterizing these errors directly from image artifacts would eliminate the need for this manual calibration, enabling reconstruction of patient studies even when calibration data are missing or outdated.

To characterize this second scenario, seven calibrated rodent cranial studies were acquired using a SEDECAL micro-CT system. Experiments were carried out in accordance with the Animal Experimentation Ethics Committee of the Community of Madrid (Ref. PROEX 332/15), following the EU Directive 2010/63EU and Recommendation 2007/526/EC, and the enforcement in Spain from RD 53/2013. Using the FUX-Sim framework (Abella et al., 2017), we simulated 25 sets of miscalibrated projections for each volume, with horizontal misalignments drawn from a random uniform distribution within a range of ±1 mm. Volumes of 256 × 256 × 200 voxels were generated using FDK-based reconstruction. The whole dataset was normalized to the ImageNet’s range to approximately match the input expected by the pretrained models. Instead of normalizing input channels independently, a single mean of 0.485 and standard deviation of 0.229 were employed throughout the entire dataset to preserve correlation between image intensities and real-world densities. Then, the volumes were partitioned into 2D axial slices. The tolerance, defined as the smallest misalignment producing artifacts noticeable to the naked eye, was set to ±0.1 mm (Abella et al., 2021). To ensure robust evaluation, the data were split by subject. One rodent study (5,000 images) was reserved as a test split and was only employed to assess generalization *a posteriori*. The remaining rodent studies were split into training (five studies, 25,000 images) and validation (one study, 5,000 images) partitions. The validation rodent study was selected at random for each subject-level partition. All models in this scenario operate on individual slices. To mitigate the influence of outliers from slices with sparse information, the misalignment prediction for each volume is defined as the median of the predictions across its constituent slices.

### 2.3 Experiments

The performance of DEL and data augmentation in the NAS framework was evaluated against predesigned architectures in the two scenarios described in Section 2.2. Our experimental design serves as an ablation study, isolating the contributions of each method before assessing their joint performance. In all cases, the optimal learning rate was approximated using Leslie Smith’s learning rate test (Smith, 2017). All models were trained using the AdamW optimizer until convergence with a patience of 8 epochs. Training convergence, DEL weighting and pruning, and NAS model selection criteria were all based on validation performance; the test sets did not influence the experimental design nor training procedures and were only employed *a posteriori* to assess the generalization capabilities of the models. NAS population sizes and search lengths were selected *ad hoc* to mimic the compute budgets expected in real-world scenarios. All Chimera Algorithm hyperparameters maintained their default values as per the rationale laid out in Section 2.1.2. We employed repeated validation partitions for every experiment to ensure generalizability and minimize bias. The number of validation images remained constant (see Section 2.2) and the number of partitions for each experiment was defined to maintain equivalent compute budgets. No data augmentation was employed unless explicitly stated.

#### 2.3.1 Transfer learning (baselines)

Four baseline configurations were established to benchmark the performance of transfer learning models of varying complexity across the two use cases:

- LeNet-5 (Lecun et al., 1998) in CIFAR-10: A shallow architecture (60,000 weights) trained on CIFAR-10 with random initialization. Following the Leslie Smith test, a learning rate of 10^−3^ was used. Performance was averaged across 32 stratified random validation partitions.
- VGG11 (Simonyan and Zisserman, 2014) in CIFAR-10: A deep architecture with over 133 million parameters. The final fully connected layer was modified for 10-class output. VGG11 was tested both with random initialization and using ImageNet-pretrained weights, with a learning rate of 10^−4^. Performance was assessed across the same 32 partitions used for LeNet-5.
- VGG11 in CT Misalignments: Adapted for regression by modifying the final layer for a single-value output and replicating grayscale input images across three channels to match expected size. Performance was assessed in two subject-level validation partitions. A cyclical learning rate (4-epoch cycle) was used. The Huber loss (Huber, 1992) was used as the loss function to limit the influence of slices with limited information during training, which may introduce considerable noise into the training process; the 𝛿 value, which controls sensitivity to outliers, was selected via grid-search within the range [0, 0.5] at 0.1 intervals. A validation-best value for VGG11 yielded 𝛿 = 0.1, which aligned with the previously defined tolerance value and was subsequently applied to all models trained on this dataset. Note that this choice also affected the fitness scores and model selection criteria for subsequent NAS searches on the CT misalignment task: as the Huber loss is more lenient towards low-performing models, the Onlooker Bees had a higher chance of selecting and exploring around them than they would have had under a pure MSE loss. This is useful for two reasons: first, most architectures in the diverse population had opportunities for improvement; second, it reduced the effects of training stochasticity and noise in the architecture space by allowing exploration around lower-performing models within high-performing clusters.
- VGG19 in CT Misalignments: Evaluated to determine if increased depth improves regression in low-sample scenarios. This model used identical architectural modifications, training hyperparameters and subject-level validation partitions as the VGG11 CT baseline to ensure a direct comparison.

#### 2.3.2 Deep ensemble learning (DEL)

To identify the most effective strategies for subsequent tests, we evaluated all combinations of data and model weighting (see Section 2.1.1) using a standalone DEL framework based on the LeNet-5 architecture and CIFAR-10 dataset. Each new learner was randomly initialized and trained on an auxiliary dataset, generated according to the specific data weighting scheme and current ensemble’s performance on the original dataset. Every ensemble was composed of 32 individual learners. Once constructed, ensembles were pruned by selecting models based on their individual validation error, reducing the final populations. Each weighting combination was evaluated across 10 stratified random validation partitions.

#### 2.3.3 Neural architecture search (NAS)

To assess the performance of NAS as a standalone method, the search space was defined by models consisting of a feature extractor composed of convolutional and pooling layers, while classifiers remained fixed. In the CIFAR-10 experiments, the classifiers contained a single fully connected layer with 10 output classes; in the CT misalignments case, they consisted of three 4,096-unit layers with ReLU and *p* = 0.5 dropout. Five experiments were conducted to compare performance against transfer learning and establish a baseline for subsequent integrated DEL-NAS tests:

- Shallow Search in CIFAR-10: The Chimera Algorithm built models from scratch. The search algorithm was run with a population size (*Np*) of 4 models and a search length of 16 iterations to evaluate the algorithm’s ability to identify high-performing architectures within relatively shallow searches. This experiment was replicated across 4 stratified random validation partitions.
- Deep Search in CIFAR-10: We increased *Np* to 8, search length to 32, and applied an exhaustion limit (*ex_max_*) of 16 to quantify performance gains over shallow runs using the same 4 partitions.
- Scratch_NAS_ in CT Misalignments: The Chimera Algorithm built models from scratch with *Np* = 6 and a search length of 16 iterations, fitting a 48-h compute budget per run. The same two subject-level validation partitions used to assess the performance of VGG11 and VGG19 were employed. A cyclical learning rate with a cycle length of 4 epochs was used. As the Leslie Smith tests did not produce stable learning rate bounds across models of varying sizes, bounds were recalculated for every new architecture. To optimize this process, the search for these bounds was restricted to an order of magnitude above and below those of the parent model, based on the assumption that minor architectural mutations would not cause significant fluctuations in the optimal learning rate.
- VGG11_NAS_ for CT Misalignments: The search was initialized in the same two partitions with four mutated copies of VGG11. Decreasing the search length to 12 iterations to maintain an equivalent compute budget, this experiment tested the benefit of starting from a predesigned baseline.
- VGG19_NAS_ for CT Misalignments: Similarly, the search was initialized in the same two partitions with four mutated copies of VGG19 over 8 iterations to match the compute budget of the previous experiments.

#### 2.3.4 Aggregated NAS populations via DEL (NAS + DEL)

Four experiments were conducted to determine how the integration of DEL within the NAS framework influence scales with task complexity and data scarcity:

- Multi-batch Gaggle in CIFAR-10: The Gaggle Algorithm generated models from scratch using 4 batches, *Np* = 4, and 16 search iterations per batch. Accuracy-based model weighting and failed-prediction data resampling were applied between batches. This experiment was conducted across four stratified random validation partitions.
- Single-batch Gaggle in CIFAR-10: A single-batch run with *Np* = 8 and search length of 32 iterations with accuracy-based weighting served as a baseline to compare the multi-batch approach against.
- Single batch Gaggle in CT Misalignments: The three populations previously generated via the Chimera Algorithm were combined into ensembles using the Gaggle Algorithm. The resulting ensembles were pruned based on the Mean Absolute Error (MAE) of the models’ median prediction for validation volumes. The resulting ensembles generated through validation-based pruning are referred to as VBP: Scratch_VBP_, VGG11_VBP_, and VGG19_VBP_.
- Oracle Upper-Bound Pruning in CT Misalignments: We analyzed all possible model combinations within the three CT populations to minimize the bias produced by the pruning strategy employed, identifying theoretically optimal configurations *a posteriori*. While models were weighted based on their validation MAE, the optimal ensembles were identified according to their test MAE. This model selection method allows us to establish performance upper bounds for the ensembles, although it is not comparable to deployable model-selection strategies as it is informed on test data. These ensembles resulting from oracle upper-bound pruning are referred to as OUB: Scratch_OUB_, VGG11_OUB_ and VGG19_OUB_.

#### 2.3.5 Data augmentation

Four experiments were conducted to assess how the addition of data augmentation (DA) schemes might influence the architectural performance landscape, shaping the effectiveness of NAS at global and local scales:

- Global CIFAR-10 DA Sensitivity Landscape: 64 randomly sampled architectures were trained with a learning rate of 10^−3^ using five DA schemes:
- DA0: Baseline (no transforms).
- DA1: Horizontal flips (*p* = 0.5) and affine transforms (rotation ± 30°, zoom *ϵ* [0.9, 1.1], shear ± 10°).
- DA2: Gaussian blurring (*σ ϵ* [0.5, 1.5] pixels), horizontal flips (*p* = 0.5), and histogram equalization (*p* = 0.5).
- DA3: PyTorch’s AutoAugment for CIFAR-10.
- DA4: Control (no transforms, different random seed) to decouple training stochasticity from augmentation effects.
- Local CIFAR-10 DA Sensitivity Landscape: the randomly sampled architecture with the best mean performance across data augmentation schemes in the CIFAR-10 dataset was selected. The five models obtained by pretraining with the five data augmentation schemes were maintained as base models. A population of 16 architectures was generated by performing a single layer mutation to this base architecture, while a second population of 16 architectures was generated via three consecutive mutations. All models were trained under the same data augmentation schemes. Non-mutated layers inherited the weights from the base models. This test was performed to check if the observed properties of the global landscape are maintained within local regions of the architecture space.
- Global CT Misalignments DA Sensitivity Landscape: 64 randomly sampled architectures were trained using:
- DA0: Baseline (no transforms).
- DA1: Additive Gaussian noise (σ ϵ [1, 500]).
- DA2: Horizontal flips (*p* = 0.5).
- DA3: Gaussian blurring (σ ϵ [0.1, 1] pixels).
- DA4: Control (no transforms, different random seed) to decouple training stochasticity from augmentation effects.
  These sets of transforms were heuristically selected as the ones that produced the most improvement over non-augmented training for VGG11. Learning rates were determined individually via the Leslie Smith test.
- Local CT Misalignments Sensitivity Landscape: Similarly to the CIFAR-10 setup, the top-performing CT architecture served as the parent for two mutation-based populations (1 and 3 mutations, 16 offspring each). Weights were inherited from the original models, and learning rates were again obtained through the Leslie Smith test.

No augmentations were applied at either the dataset or subject levels. All augmentations were applied independently for each image during training. For each experiment, the validation images were repeated ten-fold and augmented to maintain consistent validation partitions to ensure a fair comparison across all models. The test images were not augmented.

#### 2.3.6 Statistical assessment

To check whether the aggregation of NAS-generated models into ensembles outperformed the individual learners in the CT artifact scenario, we performed a three-way Analysis of Variance (ANOVA) on the Test Mean Absolute Error (MAE). The linear model was constructed hierarchically to encompass all three main factors (SLP: subject-level partition; Type: standalone learner, VBP ensemble, and OUB ensemble; and Initialization: starting the search from scratch, VGG11 or VGG19) along with their respective two-way interaction terms (SLP × Type, Initialization × Type, and SLP × Initialization). Type III Sum of Squares was employed to ensure unbiased hypothesis testing given the interaction terms and potential imbalances across group sizes. Following the primary ANOVA, post-hoc analysis was executed to explore the main effects independently of the interactions. Pairwise comparisons of the factor levels were conducted using Estimated Marginal Means (EMMs). To stringently control the family-wise error rate across multiple comparisons, *p*-values for multi-level factors (Initialization and Type) were adjusted using Tukey’s Honestly Significant Difference (HSD) method. Statistical significance was assessed at a baseline threshold of alpha = 0.05. Model diagnostics confirmed the validity of the ANOVA results: Levene’s test was non-significant (*p* > 0.05), confirming homogeneity of variances, and residual analysis demonstrated adequate normality and homoscedasticity. All computational analysis was implemented in R using the BlueSky Statistics interface (v10.3.4).

To formally assess whether individual learners and ensembles exhibit practical equivalence in the CT misalignment task, we supplemented the traditional NHST framework with a Two One-Sided Tests (TOST) procedure using the TOSTER R package. We prespecify an equivalence margin of ±100 μm, which aligns with the practical tolerance values described in Section 2.2.2, corresponding to the smallest detector misalignments that produce artifacts noticeable to the naked eye. Equivalence is concluded if the 90% confidence interval (CI) for the mean difference falls entirely within these bounds, effectively rejecting effect sizes large enough to be considered practically meaningful. To further characterize the statistical resolution of our experimental design (n_LEARNER_ = 28, n_ESEMBLE_ = 6), we complemented this analysis with a sensitivity power analysis to determine the Minimum Detectable Equivalence Margin (MDEM) the study was powered (80%) to resolve.

For the CIFAR-10 Gaggle experiments, the consistency of the search strategies was formally evaluated by comparing the performance stability across validation partitions using an F-test for equality of variances.

To assess whether the correlation between data augmentation schemes decreased significantly within local architectural clusters, a comparison of correlation coefficients has been performed. The populations generated via a single mutation and via three mutations, respectively defining clusters of relatively small and moderate sizes, have been compared with the global populations for the CIFAR-10 and the CT artifact scenarios independently (*n* = 64 for the global populations and n = 16 for the clusters).

## 3 Results

CIFAR-10 results (Table 1) confirm that combining NAS and DEL achieves superior accuracy compared to either standalone method. However, this approach remains unable to match the performance of VGG11 with ImageNet pretraining.

**Table 1:** Results of the methods proposed on the CIFAR-10 dataset.

Approach		Training % accuracy	Validation % accuracy	Test % accuracy	Compute time (h)	Number of models
Baselines	LeNet-5	68.96 ± 1.73	60.52 ± 0.76	60.52 ± 0.83	0.17 ± 0.04	1
	VGG11 (random init.)	85.43 ± 3.43	70.76 ± 0.98	70.56 ± 0.85	0.24 ± 0.03	1
	VGG11 (pretrained)	93.87 ± 1.61	84.39 ± 0.87	84.01 ± 0.61	0.23 ± 0.01	1
DEL	Acc-based weightings	80.83 ± 0.72	71.01 ± 0.57	70.83 ± 0.30	5.43 ± 0.15	25.00 ± 1.26
NAS	Pop. Size 4, search length 16	85.67 ± 2.84	74.52 ± 0.91	74.43 ± 0.97	8.04 ± 0.23	1 (5.2 ± 1.3 generated)
	Pop. Size 8, search length 32	85.07 ± 4.73	74.08 ± 4.02	73.74 ± 4.00	36.23 ± 6.06	1 (23.0 ± 4.3 generated)
NAS + DEL	Pop. Size 4, search length 16	94.87 ± 0.45	81.76 ± 0.73	81.11 ± 0.25	36.05 ± 1.78	14.75 ± 2.86
	Pop. Size 8, search length 32	94.35 ± 1.08	81.61 ± 0.84	81.05 ± 0.84	36.62 ± 5.79	19.00 ± 6.68

Experiments in this table totalized 1077.86 GPU hours to run across all partitions.

**Figure 2:**
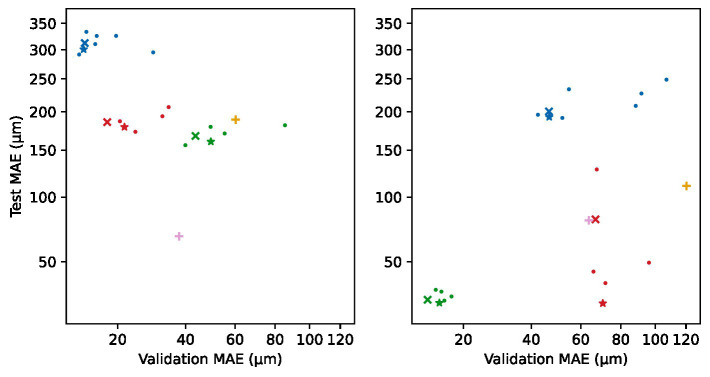
Mean Absolute Error of the models and ensembles tested on the CT artifact dataset for the first (left) and second (right) subject-level partitions. VGG11 (yellow +), VGG19 (pink +), the models generated via NAS (●), and the ensembles resulting from validation-based (×) and oracle upper-bound (★) pruning, initializing the searches from scratch (blue), from mutated copies of VGG11 (green) and mutated copies of VGG19 (red) are shown.

The results for the CT geometrical misalignment task (Figure 2 and Table 2), utilizing the MAE of volume-wise predictions as the performance metric, illustrate the performance of individual learners from each search, alongside VBP and OUB ensembles.

**Table 2:** Results of the methods proposed on the CT artifact dataset.

Approach		Subject-level partition I		Subject-level partition II	
		Validation MAE (μm)	Test MAE (μm)	Validation MAE (μm)	Test MAE (μm)
Baselines	VGG11	60.3	189.0	120.4	110.8
	VGG19	37.7	67.2	63.8	79.6
NAS	Scratch_NAS_	17.5 [12.6–43.6]	313.7 [311.1–336.9]	72.7 [30.5–61.4]	217.5 [191.5–217.4]
	VGG11_NAS_	57.6 [10.3–45.4]	171.8 [148.7–178.0]	15.6 [28.2–63.2]	32.1 [29.2–58.4]
	VGG19_NAS_	27.8 [25.0–60.1]	190.0 [173.0–202.2]	75.4 [42.9–77.9]	64.8 [53.5–82.7]
NAS + DEL (validation-based pruning)	Scratch_VBP_	13.1 [−4.8–44.25]	312.3 [299.7–340.6]	46.6 [13.1–62.1]	200.5 [180.1–221.1]
	VGG11_VBP_	43.6 [−5.3–44.4]	167.4 [138.8–180.2]	12.6 [12.5–62.2]	29.7 [19.2–60.7]
	VGG19_VBP_	17.6 [9.4–59.1]	185.7 [163.0–204.5]	67.0 [27.2–76.9]	80.4 [43.5–85.0]
NAS + DEL (oracle upper-bound pruning)	Scratch_OUB_	12.9 [−2.1–46.9]	300.7 [286.5–327.4]	46.7 [15.7–64.7]	192.7 [166.0–207.9]
	VGG11_OUB_	49.6 [−2.7–47.0]	160.3 [125.6–167.1]	14.8 [15.1–64.8]	28.3 [6.1–47.6]
	VGG19_OUB_	21.6 [12.0–61.7]	179.1 [149.9–191.3]	70.6 [29.8–79.5]	28.1 [30.3–71.8]

Values for NAS-generated models, VBP and OUB ensembles represent Mean [95% CI], derived from validation and test ANOVA residual variabilities. Experiments in this table totalized 283.52 GPU hours to run across both subject-level partitions.

Validation-based and oracle upper-bound pruning yielded distinct ensembles across all evaluated populations. The discrepancy between validation and test performance reflects a clear tendency for models to overfit the validation partition. Notably, the test-to-validation MAE ratio was approximately five times higher in the first subject-level partition than throughout the second, a divergence that underscores the importance of constructing representative partitions to ensure reliable model selection.

### 3.1 Transfer learning (baselines)

For the CIFAR-10 classification task, VGG11 outperformed LeNet-5 across training, validation, and test sets. Furthermore, inheriting weights from ImageNet pretraining significantly enhanced performance. On the CT misalignment task, VGG19 consistently outperformed VGG11. Notably lower validation and test MAE values indicate that VGG19 successfully captured complex features without succumbing to memorization.

### 3.2 Deep ensemble learning (DEL)

Detailed results for all CIFAR-10 weighting strategies are presented in Table 3. The highest performance was achieved by a narrow margin through a combination of failed-prediction data resampling and accuracy-based model weighting (in bold). Conversely, ensembles employing the loss function modification strategy (in grey) performed noticeably worse in both validation and testing.

**Table 3:** Performance of the LeNet-5 ensembles generated for the CIFAR-10 dataset using all the combinations of sample weighting and model weighting for the complete and pruned populations—C and P, respectively.

Model w.		Mean prediction			Accuracy-based weighting			Confusion matrix-based weighting		
Data w.		Train acc.	Valid. acc.	Test acc.	Train acc.	Valid. acc.	Test acc.	Train acc.	Valid. acc.	Test acc.
No weighting	C	79.73 ± 0.49	70.46 ± 0.48	70.30 ± 0.28	80.11 ± 0.73	70.74 ± 0.64	70.42 ± 0.49	79.44 ± 0.87	69.88 ± 0.54	69.87 ± 0.43
	P	80.20 ± 0.52	70.71 ± 0.45	70.45 ± 0.29	80.61 ± 0.82	70.94 ± 0.61	70.61 ± 0.46	79.79 ± 0.90	70.05 ± 0.51	70.01 ± 0.39
Failed prediction resampling	C	80.23 ± 0.78	70.75 ± 0.33	70.54 ± 0.40	80.46 ± 0.66	70.84 ± 0.61	70.68 ± 0.28	79.33 ± 0.63	69.90 ± 0.41	69.91 ± 0.43
	P	80.46 ± 0.76	70.85 ± 0.35	70.63 ± 0.38	**80.83 ± 0.72**	**71.01 ± 0.57**	**70.83 ± 0.30**	79.83 ± 0.78	70.13 ± 0.46	70.10 ± 0.42
Accuracy-based resampling	C	79.81 ± 0.55	70.40 ± 0.49	70.41 ± 0.37	80.04 ± 0.55	70.90 ± 0.51	70.48 ± 0.30	79.11 ± 0.64	70.17 ± 0.47	70.06 ± 0.35
	P	80.18 ± 0.65	70.59 ± 0.46	70.58 ± 0.32	80.35 ± 0.56	71.06 ± 0.56	70.65 ± 0.37	79.63 ± 0.58	70.36 ± 0.49	70.18 ± 0.39
Loss function modification	C	79.57 ± 1.13	66.82 ± 0.53	66.58 ± 0.43	79.93 ± 0.86	66.89 ± 0.506	66.70 ± 0.332	77.97 ± 1.38	66.96 ± 1.24	66.64 ± 1.60
	P	79.63 ± 1.10	66.90 ± 0.57	66.63 ± 0.45	79.94 ± 0.91	66.98 ± 0.55	66.71 ± 0.37	78.29 ± 1.36	67.21 ± 1.29	66.83 ± 1.56

**Figure 3:**
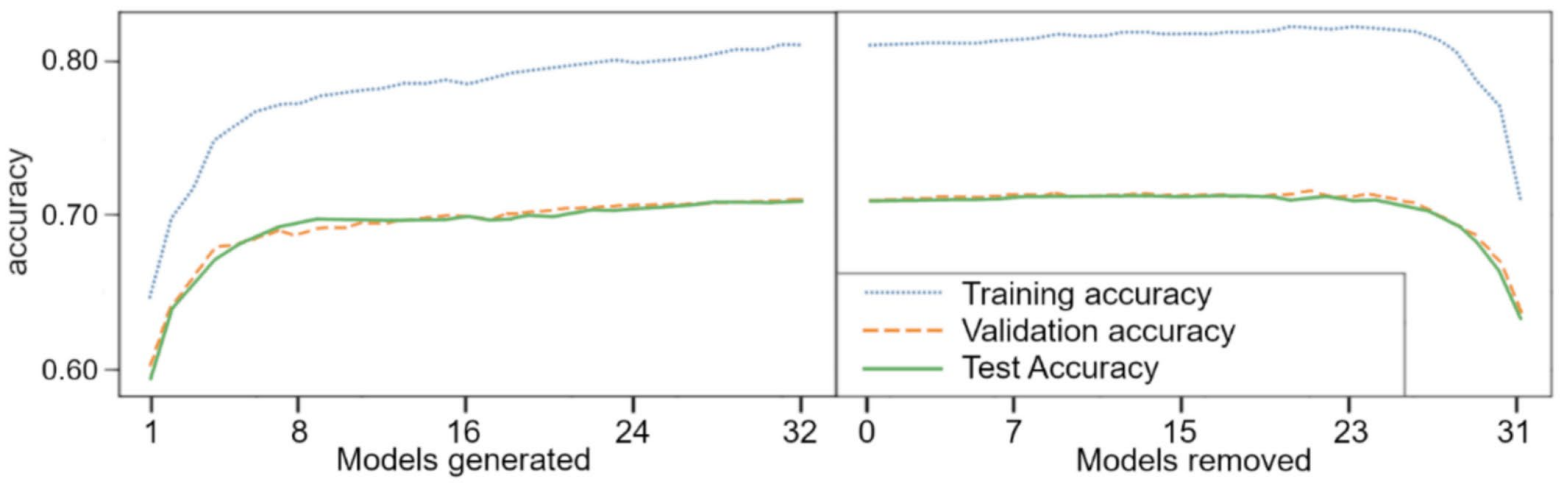
Performance of an example ensemble, adding the models by creation order (left) or removing them according to their standalone accuracies (right).

Figure 3 illustrates the behavior of a representative ensemble using accuracy-based resampling and model weighting. Ensemble accuracy increased as more models were added, though the rate of improvement diminished rapidly. Subsequent pruning further enhanced performance until the removal of essential learners caused accuracy to regress towards that of the best standalone model. In all cases, pruned populations yielded superior results compared to the original ensembles while using fewer models.

### 3.3 Neural architecture search (NAS)

Top-performing models in the CIFAR-10 NAS runs were consistently found within deep searches. However, the stochastic nature of late exhaustion and solution reinitialization introduced several low**-**performing models in the populations near the end of the searches, which reduced the overall mean population accuracy. The computational cost and number of models generated in the deep searches were approximately 4-fold that of the shallow searches, aligning with the configured batch size and search length.

For the CT misalignments task, initializing searches from VGG11 or VGG19 yielded considerably better generalization than starting from scratch. Although scratch-built models achieved competitive validation performance, their test MAE was an order of magnitude higher, indicating clear overfitting.

### 3.4 Aggregated NAS populations via DEL (NAS + DEL)

Integrating NAS with DEL on the CIFAR-10 dataset outperformed either method on its own, though none of the ensembles was able to surpass the accuracy of the pretrained VGG11 baseline. While the mean performance was nearly identical for the single- and multi-batch Gaggle approaches, a formal F-test for equality of variances confirmed that the multi-batch strategy was significantly more consistent [*F*(3,3) = 11.29, *p* = 0.039]. The Gaggle Algorithm (employing failed-prediction data resampling in between shallow Chimera searches) provided slightly more consistent results than ensembling a single protracted search.

In the CT misalignments scenario, all Gaggle-generated ensembles surpassed their base learners in validation. During testing, they outperformed their worst individual constituents but failed to improve the performance of their best standalone learner. Ensembles generated through oracle upper-bound pruning consistently outperformed those generated via validation MAE-based pruning, supporting the expected generalization gap between validation and test distributions.

### 3.5 Data augmentation

**Figure 4:**
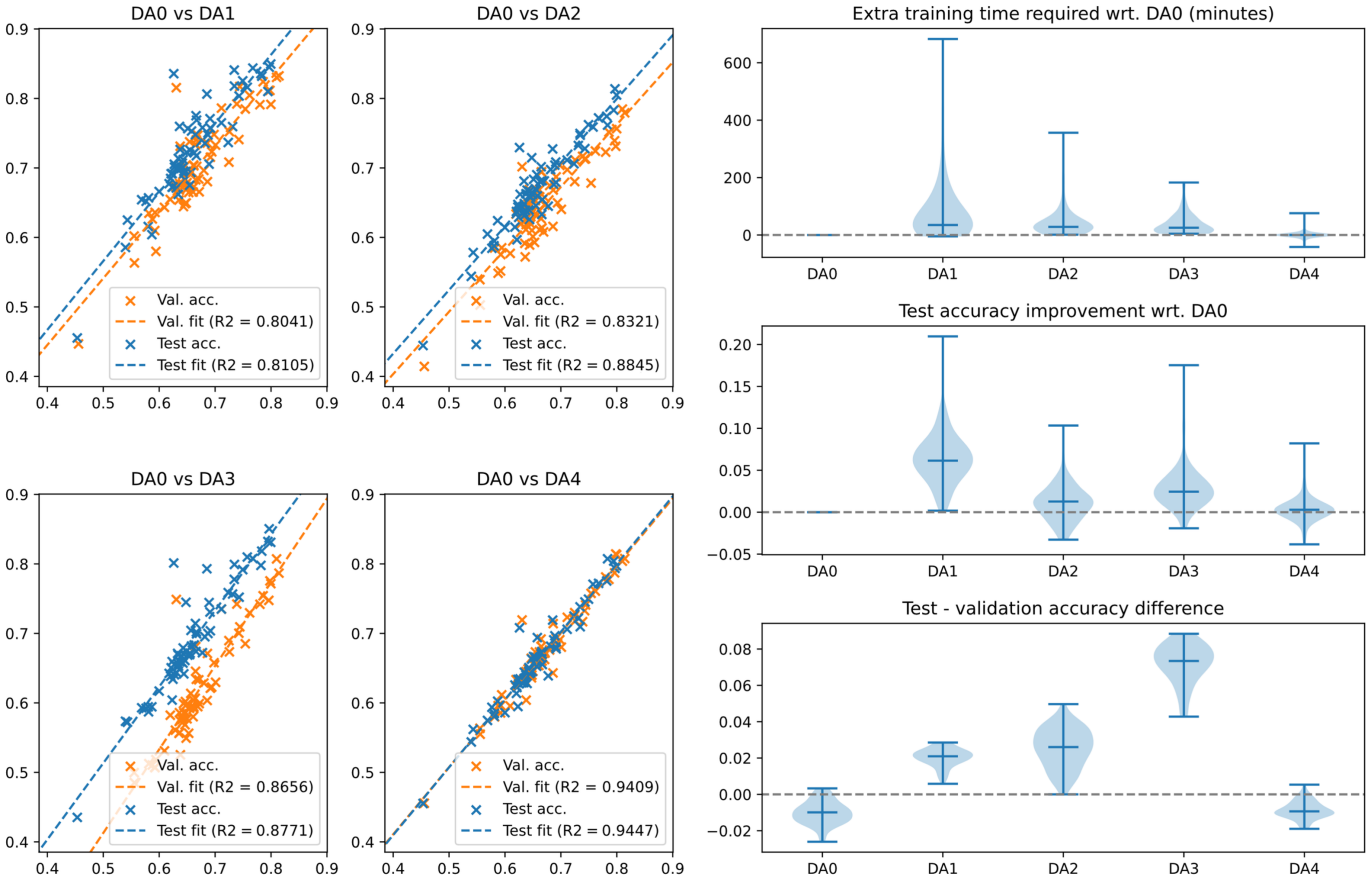
Performance comparison on the CIFAR-10 dataset of randomly sampled models using no augmentations (DA0) against geometrical transforms (DA1, top left), blurring and histogram equalization (DA2, top center), PyTorch’s AutoAugment for CIFAR10 (DA3, bottom left), and no augmentations with weight reinitialization (DA4, bottom center). Linear fits for the validation and test accuracy are shown in dashed lines. Violin plots show differences in training time (top right), test accuracy (center right) and differences in validation and test accuracy (bottom right).

Figure 4 presents the performance of randomly sampled architectures across different data augmentation schemes for CIFAR-10. DA1 yielded the best performance gains, consistently outperforming the DA0 baseline. In contrast, DA2 exhibited mixed behavior, occasionally degrading performance, while DA3 showed inconsistent results for small architectures built from scratch, aligning with expectations since PyTorch’s AutoAugment was designed for larger models. While all three data augmentation schemes increased training times and strongly correlated with non-augmented results, they introduced higher variance than that observed purely due to training stochasticity in DA4.

**Figure 5:**
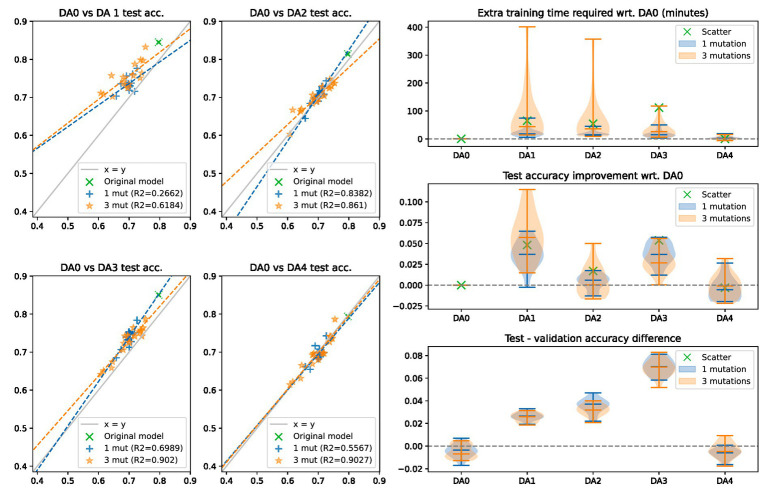
Performance comparison on the CIFAR-10 dataset of the best performing randomly generated model (green x) and models generated by mutating it once (blue crosses) and thrice (orange stars) using no augmentations (DA0) against geometrical transforms (DA1, top left), blurring and histogram equalization (DA2, top center), PyTorch’s AutoAugment for CIFAR10 (DA3, bottom left), and no augmentations with weight reinitialization (DA4, bottom center). Linear fits for the validation and test accuracy are shown in dashed lines. Violin plots show differences in training time (top right), test accuracy (center right) and differences in validation and test accuracy (bottom right).

Local neighborhood analysis (Figure 5) reveals that performance correlation depends significantly on architectural edit distance. The performance of architectures resulting from a single mutation exhibit less dispersion than that of those resulting from three mutations. However note that, despite being derivatives of the highest-performing architecture from Figure 4, no mutated offspring matched the parent’s model performance. This suggests that search or training stochasticity can yield high-performing architecture candidates whose behavior diverges significantly from their immediate structural neighbors.

Generally, data augmentation sensitivity is more consistent within single-mutation clusters than in more distant ones, as evidenced by the tighter violin plots. This indicates a direct correlation between edit distance and the variance in augmentation sensitivity. Furthermore, the correlation between different training schemes for any given architecture changes as edit distance increases. For highly similar models, the noise induced by training stochasticity appears to outweigh the performance gains attributable to minor architectural changes.

**Figure 6:**
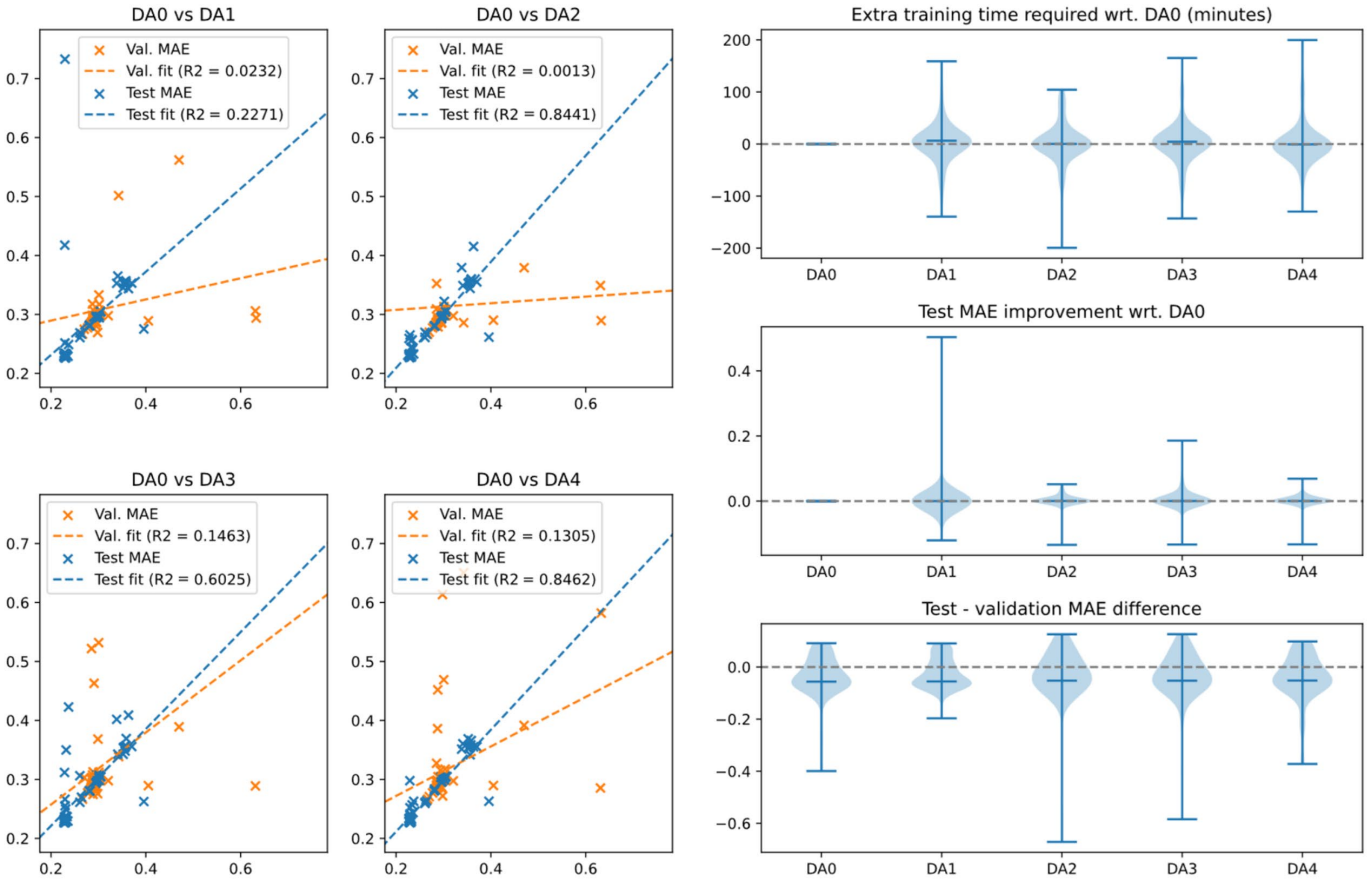
Performance comparison on the CT misalignment dataset of randomly sampled models using no augmentations (DA0) against the addition of noise (DA1, top left), horizontal flips (DA2, top center), blurring (DA3, bottom left), and no augmentations with weight reinitialization (DA4, bottom center). Linear fits for the validation and test MAE are shown in dashed lines. Violin plots show differences in training time (top right), test MAE (center right) and differences in validation and test MAE (bottom right).

The performance of randomly sampled architectures and data augmentation schemes on the CT misalignments dataset (Figure 6) diverges significantly from the CIFAR-10 results. Low correlation exists between individual training runs for each architecture, even when comparing non-augmented baselines with different seeds. This stochasticity is mirrored in the augmentation results, as no scheme consistently improved generalization. Each augmentation scheme exhibited distinct behavior: DA2 (horizontal flips) was nearly indistinguishable from simple weight reinitialization, whereas DA1 (noise) and DA3 (blurring) introduced greater complexity, exerting a more pronounced influence on generalization and yielding the top-performing models.

**Figure 7:**
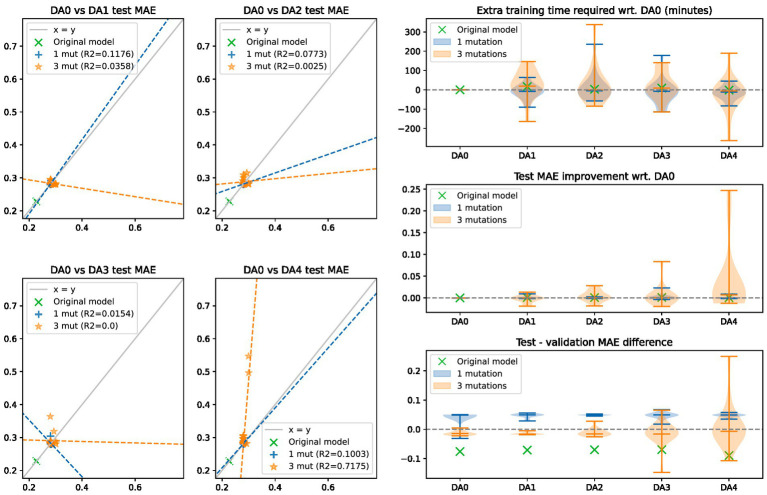
Performance comparison on the CT misalignment dataset of the best performing randomly generated model (green x) and models generated by mutating it once (blue crosses) and thrice (orange stars) using no augmentations (DA0) against the addition of noise (DA1, top left), horizontal flips (DA2, top center), blurring (DA3, bottom left), and no augmentations with weight reinitialization (DA4, bottom center). Linear fits for the validation and test MAE are shown in dashed lines. Violin plots show differences in training time (top right), test MAE (center right) and differences in validation and test MAE (bottom right).

Local neighborhood analysis (Figure 7) reveals a phenomenon similar to the CIFAR-10 scenario. Similar architectures present comparable performance, with few outliers emerging after multiple stacked mutations. Increased architectural distance correlates with higher dispersion in test error and validation-test discrepancies. Correlation between training schemes vanishes among closely related architectures, as training stochasticity eclipses gains attributable to minor architectural differences. Finally, all mutated models underperformed compared to the parent architecture, once again suggesting that the parent model represents a local optimum, identified during random sampling, with significantly higher performance than its immediate architectural neighbors.

### 3.6 Statistical assessment

Table 4 shows the results of the multi-way ANOVA model pertaining to the CT artifact dataset. Among all evaluated terms, only two main factors yielded a statistically significant effect: Initialization and SLP, while the third main factor (Type) and all two-way interactions were found to be statistically non-significant.

**Table 4:** Type III ANOVA and effect sizes.

Independent/Interaction Term	Df	Sum Sq	Mean Sq	*F* value	Pr(>F) (*p*-value)	Partial Eta^2^ (η_p_2)
Initialization	2	217689.20	108844.6	253.595	< 0.001*	0.9512
SLP	1	142850.30	142850.3	332.824	< 0.001*	0.9275
Type	2	1426.97	713.49	1.662	0.2092	0.1134
SLP × type	2	225.64	112.82	0.263	0.7709	0.0198
Initialization × type	4	561.05	140.26	0.327	0.8574	0.0479
Initialization × SLP	2	2508.78	1254.39	2.923	0.0716	0.1837
Residuals (error)	26	11159.38	429.21	—	—	—

Main factors studied are SLP (subject-level partition), Type (standalone learner, VBP ensemble, and OUB ensemble), and Initialization (starting the search from scratch, VGG11 or VGG19). *p*-values lower than 0.05 are highlighted with *.

The formal equivalence test (TOST) comparing individual learners and VBP ensembles was statistically significant at the prespecified margin of ±100 μm (*p* = 0.038). The 90% CI for the mean difference [−57.7, 93.7] μm was entirely contained within the equivalence bounds, rejecting the presence of performance differences large enough to produce artifacts noticeable to the naked eye. However, the subsequent sensitivity analysis revealed that the study’s statistical resolution (MDEM = 137 μm) was coarser than the clinical visibility threshold.

Table 5 shows pair-wise comparisons. The SLP factor, although significant (123.69 μm, *p* < 0.0001), was excluded from the *post-hoc* pairwise comparisons as its analysis does not carry any meaningful scientific interpretation regarding model ensembling. The effect of the NAS initialization was consistently significant: searching models from scratch underperforms substantially compared to either transfer learning option (*p* < 0.0001), while using VGG11 provides a small but statistically significant edge over VGG19 (*p* = 0.0104). Furthermore, the analysis regarding the effect of ensembling confirms the overall ANOVA, as no pairwise combination of model types yielded a statistically meaningful change in performance (*p* > 0.05).

**Table 5:** Statistical assessment from ANOVA analysis, comparing Initialization and type factors pairwise.

Main factor	Contrast/comparison	Mean difference (Δ, μm)	Std. Error (SE)	*t*-ratio	Adjusted *p*-value
Initialization	Search starting from Scratch vs. VGG11	157.98	7.64	20.681	< 0.0001*
	Search starting from Scratch vs. VGG19	133.81	7.64	17.516	< 0.0001*
	Search starting from VGG11 vs. VGG19	−24.17	7.64	−3.164	0.0104*
Type	Individual learners vs. VBP ensembles	−8.72	7.64	−1.142	0.4965
	Individual learners vs. OUB ensembles	−1.11	7.64	−0.145	0.9885
	VBP ensembles vs. OUB ensembles	7.61	7.64	0.996	0.5855

*p*-values lower than 0.05 are highlighted with *.

Table 6 shows the results of the comparison of correlation coefficients. We can observe that the populations generated via three mutations exhibit a data augmentation correlation closer to that of the global populations than those generated via a single mutation.

**Table 6:** Comparison of correlation coefficients results divided by dataset and data augmentation scheme pair.

Correlation comparison	CIFAR-10 dataset				CT artifact dataset			
	DA0 vs DA1	DA0 vs DA2	DA0 vs DA3	DA0 vs DA4	DA0 vs DA1	DA0 vs DA2	DA0 vs DA3	DA0 vs DA4
Global population vs. 1 mutation	*p* = 0.005*	*p* = 0.554	*p* = 0.103	*p* < 0.001*	*p* = 0.712	*p* < 0.001*	*p* = 0.026*	p < 0.001*
Global population vs. 3 mutations	*p* = 0.184	*p* = 0.746	*p* = 0.695	*p* = 0.337	*p* = 0.639	*p* < 0.001*	*p* = 0.023*	*p* = 0.266

*p*-values lower than 0.05 are highlighted with *.

## 4 Discussion and conclusions

This work empirically evaluates the synergies and antagonisms between Neural Architecture Search (NAS), Deep Ensemble Learning (DEL), and data augmentation. While these methodologies are traditionally viewed as complementary tools for improving Deep Learning performance, our results indicate that their effectiveness is fundamentally linked to the scale of the dataset and the resulting degree of model overfitting.

Our experiments on the CIFAR-10 benchmark align with existing literature (Herron et al., 2020; Chen et al., 2021), suggesting that integrating NAS with DEL (specifically via the proposed Gaggle Algorithm) outperforms standalone NAS when data partitions are representative of the target task. Moreover, prioritizing predictive performance enhanced computational efficiency. Subsampling the training dataset reduced epoch duration, while the DEL framework enabled the search to focus on very shallow models. The significantly lower variance observed in the multi-batch Gaggle runs suggests that partitioning the search into repeated batches can also mitigate the stochasticity inherent in NAS. By allowing the ensemble to ‘focus’ on misclassified samples through resampling, the Gaggle Algorithm stabilizes the training process, resulting in more reproducible performance across distinct validation partitions compared to single-search strategies. The impact of population pruning was more pronounced than in traditional DEL, as NAS-generated learners exhibited greater architectural diversity than fixed, predesigned architectures. Nevertheless, neither ensemble approach surpassed the performance of a pretrained VGG11, reinforcing that these automated methods augment, rather than substitute, the use of quality data and optimized training dynamics.

The antagonisms between NAS and DEL dominate the observed behavior in the CT misalignment task. High predictive variance across models suggests a significant selection bias, indicating that the validation partition fails to adequately characterize the underlying data distribution (Cawley and Talbot, 2010). This instability, which yields significant validation overfitting, is further evidenced by the differences in test-to-validation MAE ratios and by the variance observed across subject-level partitions, factors that directly constrain the search dynamics and optimization trajectory of the NAS process. Consistent with a more biased architecture search space, generating a great number of models increases the probability of validation overfitting. A counter-intuitive trend emerged at the same time in the comparison of predesigned architectures: despite the limited sample size, the deeper pretrained VGG19 significantly outperformed the pretrained VGG11. This suggests that, for complex biomedical artifacts or features, increased architectural depth can enhance representational capacity without necessarily inducing overfitting. Consequently, these observations indicate that in such scenarios, shorter NAS runs focused on deeper models may be more effective than protracted runs across shallower search spaces. This is consistent with the runs starting from scratch, which present the largest population sizes and search lengths, consistently resulting in the most overfit models. However, given a fixed mutation rate, deeper models would require proportionally lengthier searches to generate significant architectural alterations, requiring a careful balance between initial complexity and overfit-inducing search power. In our experiments, the populations generated from VGG11 over 12 iterations slightly but significantly outperformed those generated from VGG19 over 8 iterations, which is consistent with this requirement for balance.

Regardless of initialization and search length, ensembling NAS-generated models in this scenario consistently fails to outperform the most robust standalone learners. This occurs because the DEL framework tends to prioritize and heavily weight models with aggressive validation set overfitting, thereby diluting the contributions of architectures with superior generalization capabilities. This is supported by the statistical analysis, which showed that none of the ensembles was able to significantly outperform the expected performance of their individual learners. An overreliance on validation performance to assign the learner weights, independent of model complexity, hinders the ensemble performance in such a way that it cannot be recovered even theoretically through oracle upper-bound pruning. These findings highlight a critical need for more robust DEL weighting and pruning strategies tailored for high-overfit regimes. Specifically, readily available metrics, such as calibrated predictive uncertainty (Kuleshov et al., 2018), distribution-shift sensitivity on augmented or external data (Hendrycks and Dietterich, 2018) and sharpness-aware losses (Foret et al., 2021), have been proven to serve as meaningful reliability measures. However, their integration within the NAS pipeline as defensive regularizers to down-weight highly overfitting models and mitigate selection bias remains largely underexplored in current literature. We aim to explore these methods in future works. Nevertheless, despite sub-optimal peak performance, it is noteworthy that all ensembles consistently exhibited comparable or superior generalization to the mean performance of their individual constituents. Considering the inherent difficulty in constructing representative data partitions, leveraging DEL to reduce model variance remains a sensible approach for low-n regimes.

Our study provides an initial empirical assessment of data augmentation sensitivity landscapes. While weight loss landscapes are typically characterized as locally smooth but globally rugged, our results suggest that data augmentation sensitivity landscapes may instead be globally homogeneous yet locally stochastic. Most architectures sampled in our experiments benefit similarly from specific augmentations on a global scale but exhibit high variance within small architectural clusters. This local instability appears to be heavily task-dependent, driven by data complexity, training stochasticity and the specific synergies or antagonisms between augmentation schemes and architectural functional blocks. Even in high-n scenarios like CIFAR-10, the correlation between augmented and non-augmented training vanishes within local clusters, as reflected by the lower R^2^ scores attained. The statistical analysis carried out further supports that some correlation is lost within small local clusters, with a statistical significance inversely proportional to the cluster size. Our observation of local roughness puts the long-standing assumption that similar models derive comparable benefits from similar augmentations into perspective. Noise induced by overfitting may further hinder navigation through this landscape, limiting the efficacy of joint data augmentation and architecture optimization in complex biomedical applications. However, the observed global homogeneity suggests that a multi-fidelity approach could be a viable alternative, conducting an initial architecture exploration using simplified data augmentation schemes to identify promising architecture clusters prior to intensive joint optimization within these regions. To our knowledge, such a strategy remains unexplored, and we intend to investigate this in future work.

One limitation of our work is that we considered a single architecture optimizer applied to only two datasets which, although representative of their respective domains, may be insufficient to fully characterize the complexities of NAS dynamics. Although we achieved lower accuracies with respect to the state-of-the-art in the CIFAR-10 scenario, the main goal of this work was not to attain competitive performance but, rather, to provide a comparative assessment of DL methods and their interactions under different data regimes within constrained compute budgets expected at an average research institution. As such, we employed a varying number of random validation partitions in the CIFAR-10 scenario to ensure similar compute budgets for all experiments, although the effects of DEL, NAS and their combination are consistent and significant. It could be argued that our relatively reduced sample size may prevent us from finding significant effects of the Type factor in the CT artifact scenario. In this respect, besides being statistically non-significant (*p* > 0.20), its partial eta-squared (*η*^2^ = 0.11) represents a minor practical impact when contrasted against the primary drivers of the system, initialization and subject-level partition, which both account for massive proportions of variance (*η*^2^ > 0.9). Furthermore, post-hoc pairwise comparisons revealed that the absolute performance differences between the individual learners and the ensembles generated via either validation-based or oracle upper-bound pruning remained strictly below 9 μm. The establishment of statistical equivalence at the 100 μm margin (*p* = 0.038) and the subsequent sensitivity analysis directly address a possible concern regarding statistical power in low-sample partitions. While we can statistically reject differences large enough to be clinically visible, the fact that our Minimum Detectable Equivalence Margin (137 μm) is an order of magnitude larger than our observed performance gains formally distinguishes a ‘lack of power to detect a small effect’ from proof of no effect. This finding quantitatively illustrates the statistical fragility of NAS and ensembling in medical imaging: the ‘ensemble signal’ is dwarfed by the inherent noise of subject-level variance (SD ≈ 102 μm), making any marginal gains practically irrelevant and statistically ‘invisible’ against the primary drivers of system error.

Regarding the data augmentation experiments, the transformations were selected ad-hoc based on VGG11 performance, which biased the sampling on the augmentation space. This is exemplified by PyTorch’s AutoAugment yielding mixed results, despite being designed to optimize the augmentation policy for CIFAR-10. However, maintaining fixed sets of transforms was necessary to study the sensitivity landscapes. Using different augmentation schemes for the CIFAR-10 and CT misalignment tasks did not hinder our analysis, as all comparisons remained intra-domain. Moreover, very similar behaviors were observed across augmentation schemes (which covered geometric transformations, blurring, noise induction, etc.) across a very diverse architecture sampling pool. Finaly, the interactions between DEL, data augmentation and NAS, we decided to study them independently, as their combinations may lead to complex behaviors. Nevertheless, we carried out an analysis previously absent from the literature and demonstrated a real-world use case where the effects expected from hybrid data augmentation, DEL, and NAS optimization appear eclipsed by the noise introduced in the loss and architecture landscapes. This highlights a knowledge gap that hinders the transfer of these powerful approaches into real-world applications.

In conclusion, our findings demonstrate that both data augmentation and the combination of NAS populations via DEL consistently improve generalization, provided that the constituent models exhibit minimal overfitting. However, the statistical analysis carried out in a data-constrained regime shows that the ensembles cannot always guarantee significant improvements over their standalone learners, and even less so when compared directly to the top-performing individual models within the populations. While these methodologies offer viable pathways to enhance and automate architectural design, researchers operating in these regimes should approach the performance gains reported in standard computer vision benchmarks with caution. In real-world applications, the substantial computational overhead of NAS may not always be justified by the marginal improvements observed over well-tuned, deep predesigned architectures.

## Data Availability

The code necessary to replicate all experiments is available through https://github.com/BiiGXray/Chimera. The link to access the datasets generated for this study can also be found in the GitHub repository. The seeds employed for randomization are specified in the code itself. All tests were performed on an Intel® Core™ i7-7700 CPU and a NVIDIA® GeForce® RTX 2060 Super™ GPU.
